# A precision medicine trial of bupropion and sertraline for major depressive disorder using a biomarker-guided sequential multiple-assignment design

**DOI:** 10.1038/s44220-026-00671-z

**Published:** 2026-07-06

**Authors:** Peter Zhukovsky, Manuel Kuhn, Lauren R. Borchers, Boyu Ren, Sarah E. Woronko, Mohan Li, Choi Sze Tracy Lam, Ethan M. Zhang, Kerry J. Ressler, Brian P. Brennan, Gordana Vitaliano, Diego A. Pizzagalli

**Affiliations:** 1 Center for Depression, Anxiety, and Stress Research, McLean Hospital, Belmont, MA USA; 2 Department of Psychiatry, Harvard Medical School, Boston, MA USA; 3 Department of Psychiatry and Pharmacology, University of Toronto, Toronto, Ontario Canada; 4 Center for Addiction and Mental Health, Toronto, Ontario Canada; 5 Institute of Medical Psychology, Charité-Universitätsmedizin Berlin, Corporate Member of Freie Universität Berlin and Humboldt-Universität zu Berlin, Berlin, Germany; 6 Noel Drury, M.D. Institute for Translational Depression Discoveries, University of California, Irvine, Irvine, CA USA; 7 Departments of Psychiatry and Human Behavior, Neurobiology and Behavior, and Biomedical Engineering, University of California, Irvine, Irvine, CA USA

**Keywords:** Depression, Reward

## Abstract

Treatment for major depressive disorder (MDD) remains challenging as only 30–50% of patients respond to first-line antidepressant medications in primary care. Here we developed algorithms using predictors of response to sertraline and bupropion from a multisite study, and tested such markers in an independent, prospective clinical trial involving unmedicated individuals with MDD (NCT05537584). Leave-one-out cross-validation models achieved good performance in the training sample (area under the curve of 0.66–0.86). In the preregistered clinical trial, no significant differences in treatment outcomes emerged for those assigned a drug consistent versus inconsistent with their biomarkers. However, significant differences emerged in symptom reduction trajectories for those with positive markers for both medications (response rate: 71.4%) or either drug (65.4%) compared with those with two negative markers (42.9%). This is the first study using biobehavioral markers to prospectively guide assignment to two widely used antidepressants, yielding a 66.8% boost in response rate and providing foundations for larger personalized treatment studies of MDD.

## Abstract (web summary)

In this precision trial, Zhukovsky et al. developed algorithms to predict sertraline or bupropion response, assigning independent, medication-free patients with major depressive disorder to one drug or the other on the basis of biobehavioral precision markers validated in a prospective trial.

## Main

Despite substantial efforts, treatment for major depressive disorder (MDD) remains imprecise and, for many, ineffective, and still involves trial and error to determine the most effective approach. Findings from the Sequenced Treatment Alternatives to Relieve Depression (STAR*D) trial revealed that only ~50% of individuals with MDD responded (that is, showed ≥50% reduction in symptoms) to the selective serotonin reuptake inhibitor (SSRI) citalopram^1^, with even lower response rates in primary care (~30%), and over one-third failed to respond to two or more antidepressants^2^. To exacerbate these issues, it takes at least 4 weeks to evaluate the efficacy of an antidepressant. This can lead to lengthy treatment that is insufficient and unnecessary, thereby increasing patient morbidity, drop-outs and suicide risk.

Despite a large number of treatment options, no biomarker-based guidance for antidepressant medication assignment is currently available, which is especially problematic in light of the substantial clinical and biological heterogeneity of MDD^3,4^. Some individuals with MDD may benefit from SSRIs, while others might benefit from medications with different mechanisms of action. Several studies have identified objective markers that predict differential responses to distinct classes of antidepressants and could thus provide patients and clinicians with critical information to guide optimal treatment selection. However, although neural^5,6^, behavioral^7^ and clinical^6–8^ variables have been shown to predict SSRI response, lack of replications in independent samples and the retrospective design of prior studies present key challenges^8^. To address these challenges, prospective biomarker-guided trials are essential for testing biomarker model utility in independent samples.

Here, we aimed to first derive models predicting response to sertraline (SER) and bupropion (BUP), two widely prescribed antidepressants, in a large two-stage clinical trial (Establishing Moderators and Biosignatures of Antidepressant Response in Clinical Care (EMBARC^9^)). SER, in particular, is among the most frequently prescribed antidepressants across countries; however, a large number of individuals do not show adequate response and might thus benefit from different classes of antidepressants. Second, and critically, we aimed to prospectively test biomarker-guided treatment in a sequential multiple assignment randomized trial for depression (SMART-D), with the explicit goal of doubling the response rate to the first treatment given for depression^10^.

Using a Personalized Advantage Index analytic framework applied to the EMBARC dataset, we previously reported that higher depression severity, higher neuroticism, older age, less impairment in cognitive control (assessed as reduced interference effect in the Flanker task), and being employed specifically predicted positive response to SER (over placebo)^7^. This modeling framework compares predicted depressive symptoms under two different treatment scenarios, predicting response to actual treatment received and a counterfactual treatment based on patients’ pretreatment markers, with the predicted advantage quantified as the difference between the optimal and nonoptimal treatment. Conversely, we found that higher resting-state functional connectivity (rsFC) between the nucleus accumbens (NAcc) and rostral anterior cingulate cortex (rACC), stronger response bias toward a more frequently rewarded stimulus, and higher reward sensitivity (the latter two variables derived from the Probabilistic Reward Task (PRT)) specifically predicted response to BUP after failing 8-week treatment with SER^5^. Moreover, in analyses in preparation for the current prospective, biomarker-guided randomized clinical trial, we discovered that relatively increased NAcc–rACC rsFC predicted strong antidepressant response to BUP but poor response to SER (see Table 1 for demographic and clinical data for the EMBARC and SMART-D samples).

**Table 1:** Demographic and clinical characteristics of the EMBARC and SMART-D samples

	EMBARC	SMART-D	Statistic
	*n* = 103	*n* = 48	
	*n* (%) or mean (s.d.)	*n* (%) or mean (s.d.)	
Biological sex (female as reference)	75 (72.8%)	34 (70.8%)	*χ*^2^(1) = 0.00, *P* = 0.954
Age, years	37.06 (13.76)	28.98 (8.03)	*t*(141.5) = 4.53, *P* < 0.001
Race (white as reference)^a^	32 (31.1%)	21 (43.8%)	*χ*^2^(1) = 1.08, *P* = 0.299
Marital status (married as reference)^b^	18 (17.5%)	7 (14.6%)	*χ*^2^(1) = 0.00, *P* = 1.000
Education (completed higher education as reference)^b^	14 (13.6%)	31 (64.6%)	*χ*^2^(1) = 49.65, *P* < 0.001
SHAPS total	33.73 (5.66)	34.67 (4.81)	*t*(106.6) = −1.05, *P* = 0.294
NAcc–rACC rsFC^BUP and SER marker^	0.15 (0.13)	0.21 (0.20)	*t*(65.3) = −1.80, *P* = 0.076
Response bias^BUP markerc^	0.08 (0.15)	0.15 (0.13)	*t*(67.3) = −2.00, *P* = 0.050
Reward sensitivity^BUP markerc^	0.62 (0.31)	0.72 (0.42)	*t*(82.00) = −1.31, *P* = 0.194
HRSD total^SER marker^	18.40 (4.44)	17.69 (3.47)	*t*(115.0) = 1.07, *P* = 0.287
Interference Flanker^SER markera^	0.20 (0.11)	0.25 (0.20)	*t*(61.3) = −1.72, *P* = 0.091
Employment status (employed as reference)	58 (56.3%)	33 (68.8%)	*χ*^2^(1) = 1.63, *P* = 0.202
Neuroticism^SER marker^	35.49 (5.84)	34.65 (6.62)	*t*(82.2) = 0.75, *P* = 0.454

^a^Seven patients with missing observations for race and interference Flanker in EMBARC.

^b^Seven patients with missing observations for marital status and education in SMART-D.

^c^Sixty-seven patients with missing observations of response bias and reward sensitivity in EMBARC, as these two variables were only available in the second phase.

Reported statistics test for differences in the distribution of each characteristic between the two datasets. Continuous variables were compared using two-sided *t*-tests; categorical variables were compared using chi-squared tests. One SMART-D participant dropped out before completing 1 week of treatment.

Capitalizing on these prior findings, we designed a prospective study in which participants completed functional magnetic resonance imaging (fMRI) and clinical and cognitive assessments (Flanker task and PRT). Within 24–72 h, we ascertained biomarker status (BUP^+^ versus BUP^−^, SER^+^ versus SER^−^) for each participant before randomizing them into treatment. Using a double-blind, randomized design, each participant received a drug that was either consistent (that is, BUP for BUP^+^ participants, SER for SER^+^ participants) or inconsistent with their biomarker (that is, BUP for BUP^−^ participants, SER for SER^−^ participants). While other prospective biomarker-guided studies have been conducted recently^11–13^, our trial is unique in testing multimodal markers for two widely used antidepressants in a prospective double-blind implementation. We hypothesized that participants receiving treatment consistent with their biomarker would show significantly higher response rates and greater decreases in depressive symptoms compared with those receiving the drug inconsistent with their marker. We also tested whether participants who were predicted to not respond to either drug (individuals with BUP^−^/SER^−^) would show worse clinical outcomes compared with those with one or two positive markers. The primary outcome was the change in depression severity from pretreatment baseline to post-treatment across the 8-week trial. We found that, relative to patients with two negative markers, those with one or two markers were characterized by significantly larger reduction in depressive symptoms, showing that biomarker-guided treatment selection can boost efficacy for two of the most widely prescribed antidepressants around the world.

## Results

### Predictive biomarker model

On the basis of our prior findings^5,7^ derived from the EMBARC study, a treatment-assignment algorithm was developed that generated, for each patient, two marker-based indications—one for BUP and one for SER. The predictive model achieved a cross-validated area under the curve (AUC) of 0.86 for BUP and AUC of 0.66 for SER.

### BUP model

**Fig. 1:**
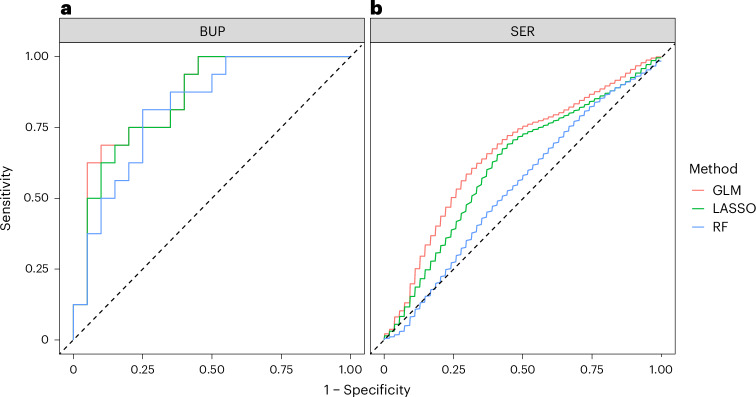
Depression response model prediction in EMBARC data. **a**,**b**, ROC curves for three candidate models when predicting response status for BUP (**a**) and SER (**b**) are shown. Specifically, we used a logistic regression without regularization (GLM), a logistic regression with LASSO penalty (LASSO) and a random forest model (RF).

Among the three candidate models, standard logistic regression achieved the highest leave-one-out cross-validation AUC (0.86), followed by least absolute shrinkage and selection operator (LASSO) logistic regression (AUC of 0.84) and random forest (RF) (AUC of 0.83). The corresponding receiver operating characteristic (ROC) curves are shown in Fig. 1a. On the basis of this comparison, a standard logistic regression model was fit to the full EMBARC stage 2 dataset (second 8-week treatment after failing 8-week treatment with SER) to obtain the predictive biomarker model for BUP response. The estimated logistic regression coefficients are reported in Supplementary Table 1a, and standardized coefficients reflecting relative contributions of each predictor are summarized in Supplementary Table 1b. For BUP, the fMRI imaging marker (NAcc–rACC resting fMRI connectivity) had the largest standardized regression coefficient, with other regressors being of a smaller but comparable magnitude. For SER, employment status showed the largest regression coefficient, with smaller but comparable regression coefficients for the other predictors of response. Baseline depression severity was positively associated with response to SER. The changes in model performance (AUC) when leaving out each predictor in Supplementary Table 2 are consistent with the standardized regression coefficient magnitudes. An optimal probability cutoff of 0.434 was identified for converting predicted probabilities into binary biomarker status.

#### SER model

Standard logistic regression also performed best for this marker, with the highest average tenfold cross-validation (CV) AUC (0.66), compared with LASSO logistic regression (AUC of 0.61) and RF (AUC of 0.56); ROC curves are shown in Fig. 1b. A standard logistic regression model was therefore fit to the full EMBARC stage 1 dataset (first 8-week treatment with SER versus placebo) to estimate the SER predictive biomarker model, with coefficients provided in Supplementary Table 1a. The optimal probability cutoff for defining binary biomarker status was 0.386.

#### Composition of marker status

On the basis of the marker prediction models and the associated probability cutoffs, the marker status among patients in EMBARC stage 2 is summarized in Supplementary Table 3. Among the 74 EMBARC participants with complete data for predicting SER and BUP marker status, 39.2% did not have a definitive marker status, most of whom were ‘double-negative’, that is, those predicted to respond to neither treatment. Overall, SER^+^ was more common than BUP^+^, with 52.7% classified as SER^+^ compared with 29.7% classified as BUP^+^.

### SMART-D trial

**Fig. 2:**
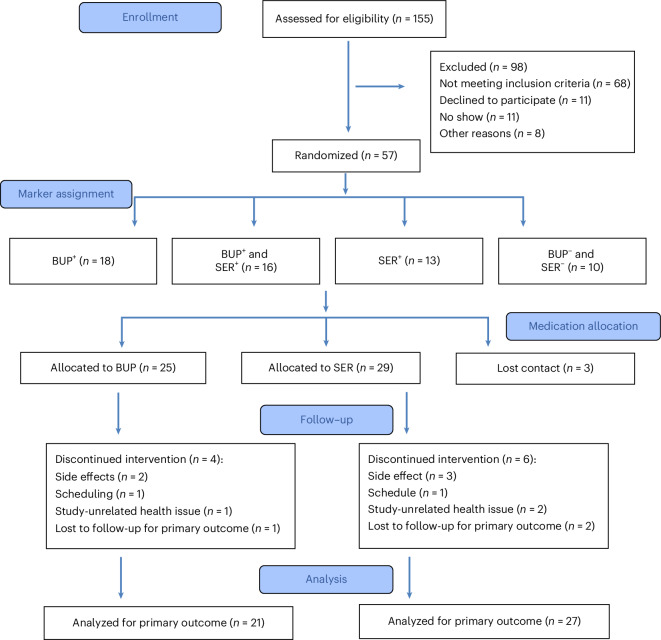
Overview of participant flow. A CONSORT diagram of participant flow in the SMART-D trial.

An overview of the participant flow in the prospective clinical trial is shown in Fig. 2, and sample demographics are summarized in Table 2. Overall, 47 participants completed at least 1 week of treatment and had sufficient information to determine baseline biomarker status; they were included in the main intent-to-treat analyses. In addition, 41 participants completed the full 8-week treatment course, and 37 of the 41 participants completed a follow-up fMRI scan.

**Table 2:** Demographic and clinical characteristics of the SMART-D subgroups

	Group 1	Group 2	Group 3	Group 4	Statistic
	*N* = 14	*N* = 17	*N* = 10	*N* = 7	
	BUP^+^/SER^+^	BUP^+^/SER^−^	BUP^−^/SER^+^	BUP^−^/SER^−^	
	*n* (%) or mean (s.d.)	*n* (%) or mean (s.d.)	*n* (%) or mean (s.d.)	*n* (%) or mean (s.d.)	
Demographics					
Biological sex (female as reference)	11 (78.6%)	14 (82.4%)	5 (50.0%)	4 (57.1%)	*χ*^2^(3) = 4.2, *P* = 0.237
Age, years	31.71 (9.74)	28.65 (7.10)	30.20 (7.63)	22.57 (3.15)	*F*(3,44) = 2.3, *P* = 0.092
Race (white as reference)	9 (64.3%)	9 (52.9%)	5 (50.0%)	4 (57.1%)	*χ*^2^(12) = 10.21, *P* = 0.598
Marital status (single as reference)^a^	10 (71.4%)	10 (58.8%)	4 (40.0%)	6 (85.7%)	*χ*^2^(9) = 7.87, *P* = 0.548
Education (completed higher education as reference)^a^	12 (85.7%)	8 (47.1%)	6 (60.0%)	5 (71.4%)	*χ*^2^(3) = 5.01, *P* = 0.171
Treatment information					
Medication (BUP as reference)	7 (50.0%)	7 (41.2%)	5 (50.0%)	2 (28.6%)	*χ*^2^(3) = 1.1, *P* = 0.781
Marker variables					
SHAPS total (baseline)	33.21 (4.69)	35.88 (4.28)	34.80 (5.63)	34.80 (5.63)	*F*(3,44) = 0.78, *P* = 0.509
NAcc–rACC rsFC^BUP and SER marker^	0.30 (0.11)	0.34 (0.12)	−0.07 (0.12)	0.09 (0.07)	*F*(3,44) = 31.0, *P* < 0.001
Response bias^BUP marker^	0.21 (0.18)	0.12 (0.12)	0.14 (0.06)	0.11 (0.07)	*F*(3,44) = 1.5, *P*= 0.239
Reward sensitivity^BUP marker^	1.03 (0.54)	0.69 (0.28)	0.56 (0.23)	0.43 (0.28)	*F*(3,44) = 5.61, *P* = 0.002
HRSD total^SER marker^	20.21 (4.10)	16.71 (2.71)	16.50 (2.76)	16.71 (2.43)	*F*(3,44) = 4.22, *P*= 0.011
Interference Flanker^SER marker^	0.09 (0.12)	0.34 (0.17)	0.22 (0.19)	0.40 (0.22)	*F*(3,44) = 7.77, *P* < 0.001
Employment status^SER marker^ (reference employed)	14 (100%)	9 (52.9%)	8 (80.0%)	2 (28.6%)	*χ*^2^(3) = 14.19, *P* = 0.003
Neuroticism^SER marker^	35.00 (9.16)	34.24 (5.18)	34.20 (5.57)	35.57 (6.43)	*F*(3,44) = 0.09, *P* = 0.965

^a^Seven patients with missing data for marital status and education.

The intent-to-treat analysis included participants who completed at least 1 week of treatment, with 1 of the 48 participants dropping out before completing their first longitudinal assessment. Reported statistics tests were used to assess differences in the distribution of each characteristic between the subgroups. Continuous variables were compared using one-way analysis of variance (*F*-test); categorical variables were compared using chi-squared tests.

### Comparison of EMBARC and SMART-D participants

We compared the EMBARC and SMART-D samples. The two studies did not differ in the proportion of biological sex, race, marital status, baseline anhedonia (Snaith Hamilton Pleasure Scale (SHAPS) scores) or any of the BUP or SER classifiers (that is, NAcc–rACC rsFC, response bias, reward sensitivity, Hamilton Rating Scale for Depression (HRSD) scores, Flanker interference effect, employment status or neuroticism; Table 1). However, on average, the EMBARC sample was older and had fewer participants who completed higher education.

### Comparison of enrolled and non-enrolled participants

There were no group differences between participants who enrolled in the study and those who did not (owing to ineligibility, loss of contact or declining to participate). Specifically, there were no statistically significant differences in biological sex (*χ*^2^(1) = 0.39, *P* = 0.533), age (*t*(113.52) = −0.07, *P* = 0.947) or the SHAPS during the screening assessment (*t*(121.88) = 1.85, *P* = 0.067).

### Demographic and clinical characteristics of SMART-D participants

We describe the SMART-D participants in Table 2. Across the sample, there were no statistically significant differences across the four groups in biological sex, age, race, marital status, education or medication assignment. There were also no group differences in baseline anhedonia, response bias, neuroticism or concomitant psychotherapy (Supplementary Table 4). There were differences in NAcc–rACC rsFC, reward sensitivity, baseline depression scores, interference scores on the Flanker and employment status, which was expected given that these variables were used to assign the respective treatment marker.

### Overall depression outcomes

Using a linear mixed-effects model (LMM) with subject-level random intercepts and random slopes by time, we found a significant reduction in Montgomery–Åsberg Depression Rating Scale (MADRS) scores quantifying depressive symptoms between pre- and post-treatment (main effect of time *t*(307) = −10.83, *P* < 0.001), showing strong effect sizes (Cohen’s *d* = −1.55, *β* = −2.12, 95% confidence interval (CI) −2.51 to −1.74). In the main intent-to-treat analysis including participants who completed at least 1 week of treatment, we also found a high response rate of 63.8% (30/47) defined as a ≥50% improvement in MADRS score from baseline and a high remission rate of 53.2% (25/47), defined by a MADRS score of ≤10 at the end of treatment. Among participants who completed 8 weeks of treatment, we found even higher response (70.7%, 29/41) and remission rates (63.4%, 26/41).

### Markers-based depression outcomes

While the original marker models derived from the EMBARC study were specific to BUP and SER, we found a large proportion of SMART-D participants to have both markers (BUP^+^/SER^+^, 29.8%, 14/47) or neither marker (BUP^−^/SER^−^, 14.9%, 7/47), with 55.3% (26/47) of participants showing one marker.

**Fig. 3:**
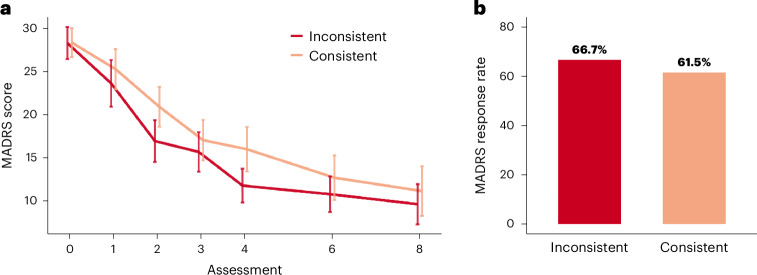
Depression symptom trajectories in participants receiving drugs that were inconsistent versus consistent with their biomarkers. **a**,**b**, Trajectories of group-specific average (±1.96 × s.e.m.) depression scores (**a**) and response rates (**b**) for participants assigned a drug consistent versus inconsistent with their biomarker indication. Mean MADRS scores are shown at each assessment with 95% CIs based on all observations (**a**). In the inconsistent group, *n* = 21 at assessments 0 and 1, and *n* = 19 at all subsequent assessments. In the consistent group, *n* = 26 at assessments 0 and 1, *n* = 23 at assessment 2, *n* = 21 at assessments 3 and 4, *n* = 20 at assessment 6, and *n* = 19 at assessment 8.

We found no significant group differences in MADRS trajectories over time according to the drug participants received (time × BUP or SER group, *F*(1,42.14) = 0.82, *P* = 0.37). Response rates were not significantly different between patients receiving SER (68.0%, 17/25) versus BUP (61.9%, 13/21) (*X*^2^ = 0.06, *P* = 0.81). MADRS depression scores declined across groups, as shown in Supplementary Fig. 1. In testing our preregistered hypothesis, we found no significant group difference in trajectories of MADRS change for those who were assigned a drug that was consistent versus inconsistent with their biomarker profile (time × consistent/inconsistent group, *F*(1,38.18) = 0.03, *P* = 0.87; Fig. 3). Response rates were not significantly different between inconsistent (66.7%, 14/21) versus consistent groups (61.5%, 16/26) (*X*^2^ = 0.13, *P* = 0.72). Remission rates also did not significantly differ between consistent versus inconsistent or BUP versus SER groups (*P* > 0.05).

**Fig. 4:**
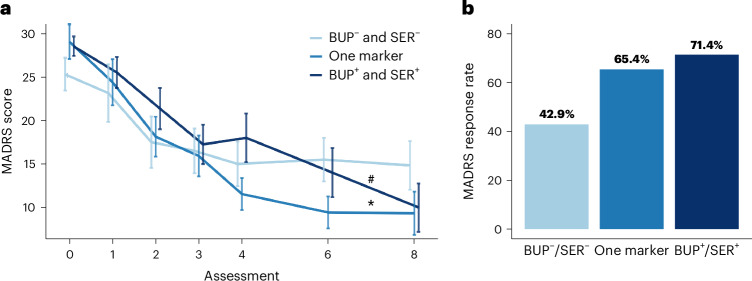
Depression symptom trajectories in participants with different biomarker status. **a**,**b**, Trajectories of group-specific average (±1.96 × s.e.m.) depression scores (**a**) and response rates (**b**) for patients with two markers, one marker or neither marker. Mean MADRS scores are shown at each assessment with 95% CIs based on all observations (**a**). In the BUP^−^/SER^−^ group, *n* = 7 at assessments 0 and 1, and *n* = 6 at all subsequent assessments. In the one-marker group, *n* = 26 at assessments 0 and 1, *n* = 22 at assessments 2–4 and *n* = 21 at assessments 6 and 8. In the BUP^+^/SER^+^ group, *n* = 14 at assessments 0–2, *n* = 12 at assessments 3, 4 and 6, and *n* = 11 at assessment 8. Significance: two-tailed *P = 0.019, one marker compared with BUP^−^/SER^−^; two-tailed ^#^*P* = 0.08, BUP^+^/SER^+^ compared with BUP^−^/SER^−^. Significance was determined using a mixed-effects model testing for time × marker group interaction.

We next tested the hypothesis that, regardless of which drug a participant received, those with neither marker (BUP^−^/SER^−^) would show worse treatment outcomes compared with those with only one marker or both markers following the preregistered analysis protocol (https://osf.io/tfrz5/). Critically, we found a significant difference in MADRS trajectories between marker groups (time × group *F*(2,38.32) = 3.85, *P* = 0.029). As expected, patients with two negative markers showed the smallest reduction in depressive symptoms regardless of drug assignment (Fig. 4), with greater reductions seen in participants with both markers (*β* = −1.037, 95% CI −2.17 to 0.100, *t*(38.07) = −1.80, *P* = 0.080) and in those with one marker only (*β* = −1.295, 95% CI −2.335 to −0.256, *t*(37.59) = −2.44, *P* = 0.019). A follow-up analysis using the four groups defined directly by the BUP and SER marker status, rather than the number of positive markers, yielded similar patterns. Specifically, both the BUP^−^/SER^+^ and BUP^+^/SER^−^ groups showed significantly higher rates of reduction in MADRS score relative to the BUP^−^/SER^−^ group (Supplementary Table 5 and Supplementary Fig. 2). Response rates were lowest in those with two negative markers (42.9%, 3/7) compared with those with one (65.4%, 17/26) or two positive markers (71.4%, 10/14), although this difference was not significant (*X*^2^ = 1.71, Fisher’s exact *P* = 0.45, Phi coefficient = 0.191) despite a medium effect size. Similarly, remission rates were lower in those with two negative markers (42.9%, 3/7) compared with those with one (53.9%,14/26) or two positive markers (57.1%, 8/14), although this difference was not significant (*X*^2^ = 0.39, Fisher’s *P* = 0.85, Phi coefficient of 0.091), which corresponds to a weak–moderate effect size.

## Discussion

In the current prospective, biomarker-guided treatment study, we first derived biomarker models predicting response to SER and BUP from the large multisite EMBARC study. Capitalizing on these independent findings, we tested whether biomarker-guided treatment selection boosted antidepressant response in the prospective SMART-D trial. This trial was carried out as part of the Wellcome Leap Multi-Channel Psych Program effort to double the number of people who respond to the first treatment they try for depression, via the integration of multimodal biomarkers (https://wellcomeleap.org/mcpsych/). While the markers showed high levels of performance in leave-one-out cross-validation, the primary analyses showed no significant differences in depression outcomes between participants who received drugs that were consistent or inconsistent with their biomarkers, possibly due to limited power to detect moderate effects. However, we did find that participants who were biomarker negative for both drugs showed significantly worse depression symptom trajectories compared with those with at least one positive biomarker, and for those with both positive markers, regardless of which drug they actually received. While response rates were almost double (66.4% higher) among those with both markers (71.4% response rate) relative to those without any marker (42.9% response rate), with those with at least one marker showing intermediate response rate (65.4%) and the effect size of this difference being in the medium range, the contingency table test did not reach statistical significance due to sample size limitations.

Our findings point to a shared biomarker signal predicting response across SER and BUP that shows a considerable degree of generalizability from EMBARC to a fully independent prospective SMART-D trial. Our markers were a combination of resting-state fMRI connectivity of NAcc to the rACC, reward learning and sensitivity, cognitive control, clinical variables (depression severity and neuroticism) and demographic variables (employment status). We found that both in the training sample (Supplementary Table 3) and in the SMART-D trial, a substantial proportion of participants were positive for both BUP and SER markers, and also a substantial proportion of participants who were negative for both BUP and SER, suggesting some shared prediction of response to either drug. SER is thought to exert therapeutic effects by inhibiting the reuptake of serotonin and thus increasing serotonergic neurotransmission, while BUP is thought to inhibit the reuptake of dopamine and norepinephrine, thus increasing signaling in these pathways. Despite differences in mechanisms of action, they both act on monoaminergic pathways. They also show similar efficacy rates (both in the current SMART-D trial and in meta-analyses^14^), thus suggesting some shared signal. Our findings are consistent with a role for reward processing and cognitive control, both processes mediated by monoaminergic signaling in the brain^15–17^, as markers of treatment responsiveness for monoaminergic medication. Future work should clarify how markers predicting response to monoaminergic medication differ from markers predicting response to brain stimulation or rapid-acting therapies, including ketamine and psychedelics.

Interestingly, we did not find significant differences in depression symptom trajectories between participants who received the drug that was indicated by their marker and those who received the drug for which they were biomarker negative, although these analyses probably had insufficient power when considering four subgroups. However, a two-group split into consistent versus inconsistent assignment also revealed no significant group differences in depression outcomes, suggesting that our markers do not differentiate well between SER and BUP.

Our results should be interpreted in light of certain limitations. First, markers were derived from EMBARC, a multisite, two-stage clinical trial comparing SER versus placebo in stage 1 and offering BUP to stage 1 nonresponders in stage 2. While it is one of the largest placebo-controlled clinical trials of its kind, the sample size, including magnetic resonance imaging (MRI), was somewhat limited. Future biomarker studies could combine data across several clinical trials to build robust biomarker models with comprehensive discovery analyses. Second, the prospective SMART-D trial included usable data from 48 participants, limiting available power to detect effects. It is likely that greater drug-specific differentiation would emerge in larger sample sizes. Third, while the resting-state fMRI measure was critical in our model (in terms of explained variance), we acknowledge the somewhat limited scalability of fMRI. Further challenge to scalability is tied to a possible need of harmonizing data processing^18^, as we used the same pipelines to build the predictive models and to identify markers during prospective randomization and were able to achieve similar biomarker distribution. Data harmonization and cross-trial generalizability have been a challenge in previous studies^19^, and we did observe higher response rates in SMART-D compared with EMBARC.

Overall, we present findings from the first prospective, biomarker-guided treatment study of two widely prescribed monoaminergic antidepressant medications. Our results suggest that we could boost response rate by using two sets of biomarkers previously identified in the EMBARC study, making an important contribution to advancing the goals of precision psychiatry. Ultimately, we strongly hope these advances will enable personalized treatment guidance to accelerate and boost antidepressant benefits.

## Methods

All research conducted here was approved by the Mass General Brigham Healthcare Institutional Review Board. EMBARC is a two-stage trial that recruited participants with MDD at four sites across the USA between August 2011 and December 2015. Institutional review boards at each EMBARC site provided ethical approval, with participants providing written, informed consent for all study procedures and being compensated for their time.

### EMBARC modeling approach

We summarize the approach used to develop prediction models for the BUP and SER markers, which identify patients’ response to each medication; additional details are provided in Supplementary Information.

#### BUP marker

Prior EMBARC results showed that pretreatment reward learning (PRT), reward sensitivity (PRT) and the rsFC of NAcc–medial prefrontal cortex, specifically the rostral ACC subdivision, predict response to BUP following SER nonresponse. Using EMBARC stage 2 data (*N* = 36), we trained prediction models on the basis of these three variables. Candidate models included logistic regression, LASSO-penalized logistic regression and RF, with performance evaluated by leave-one-out cross-validated area under the receiver operating characteristic curve. The best-performing model was refit on the full dataset. Predicted probabilities were dichotomized using an ROC-based optimal threshold to define response status.

#### SER marker

Using EMBARC stage 1 SER-treated participants (*N* = 96), we applied an analogous pipeline with five pretreatment variables: depression severity, neuroticism, cognitive control (Eriksen Flanker task), employment status and NAcc–medial prefrontal cortex rsFC. Models were evaluated using repeated tenfold CV (1,000 repetitions), and the final model was selected on the basis of mean area under the receiver operating characteristic curve. The classification threshold was determined from the cross-validated ROC curve.

### Overview of the SMART-D trial design and participants

The SMART-D study is a single-site, prospective, biomarker-guided, precision double-blind medicine trial that tested clinical, neural and cognitive outcomes in unmedicated participants with MDD who were randomly assigned SER or BUP on the basis of their biomarkers. SMART-D was registered on ClinicalTrials.gov (NCT05537584) and carried out at McLean Hospital, Belmont, MA, USA. Participants were enrolled between 27 October 2022 and 26 August 2025. All participants provided written informed consent after the procedures had been fully explained in accordance with the Helsinki guidelines, under the approval of the Mass General Brigham Healthcare Institutional Review Board, and were compensated for their time. Treatment-seeking individuals were recruited from the community using word of mouth, social media, public transport advertisements and BuildClinical recruitment services.

Participants who passed a screening session and a pretreatment assessment electrocardiogram (ECG) screening then completed MRI, electroencephalogram (EEG) and cognitive and clinical assessments alongside blood sampling to ascertain their biomarkers for BUP and SER. If a participant’s ECG was deemed normal, they were randomized to receive medication that was either consistent (50%) or inconsistent (50%) with their biomarkers and completed 8 weeks of treatment. Data required for the biomarker determination were processed by team members blind to treatment assignment; biomarker determination was carried out by a biostatistician (B.R.), who was also blind to treatment assignment; the biostatistician then provided the information about biomarker status to the McLean Research Pharmacy, which assigned 50% of the BUP-positive patients to BUP and 50% to SER (and vice versa for the SER-positive patients). Randomization was stratified by Flanker interference, employment, neuroticism, HRSD, PRT response bias and reward sensitivity and accumbens–rACC connectivity.

Participants met with one of two study psychiatrists (B.P.B. or G.V.) at the initial consent session, and then at weekly intervals during treatment to discuss medications, side effects and treatment progress for a total of eight sessions. During weeks 5 and 7, no psychiatrist meeting was conducted. Finally, patients completed a post-treatment assessment, including MRI, EEG and cognitive and clinical assessments.

We analyzed data from 48 participants; 47 participants completed at least 1 week of treatment, and 1 participant completed the baseline assessment and was included in the mixed-effects models. Of these 48 participants, 41 completed the full 8 weeks of treatment. We analyzed the differences between consistent versus inconsistent assignment and between biomarker load (SER^−^/BUP^−^ versus SER^+^/BUP^−^ and SER^−^/BUP^+^ versus SER^+^/BUP^+^) on our primary outcome of depression severity (MADRS scores).

### Eligibility criteria

Eligibility was determined at the screening session. To be eligible, participants had to meet criteria for a current diagnosis of MDD based on the Diagnostic and Statistical Manual of Mental Disorders, Fifth Edition, Text Revision and Structured Clinical Interview for DSM-5 assessed by a masters- or PhD-level clinician, and have elevated depression (HRSD >15) and anhedonia (SHAPS >24) scores. Participants’ treatment resistance was assessed using the Antidepressant Treatment Response Questionnaire^20^, and they were excluded if they did not respond to both BUP and SER in the current MDD episode or if they did not respond to more than three adequate antidepressant or therapy treatment trials. Participants were permitted to engage in psychotherapy provided that treatment had been initiated before study enrollment and remained stable throughout participation (that is, no changes in treatment type or frequency); participants were told that initiation of a new psychological treatments or modification of existing treatment plans during the study would lead to study discontinuation. A total of 14 participants were receiving concomitant psychotherapy, with no group differences (Supplementary Table 4). Participants were required to have no contraindications to the study drugs. Other exclusion criteria were significant medical conditions, including neurological, psychiatric or substance use disorder comorbidities (generalized anxiety disorder, specific phobia and social anxiety disorder was allowed, but MDD had to be the principal complaint), and MRI contraindications. This trial included adults aged between 18 years and 64 years. Recreational use of alcohol and cannabis was not an exclusion criterion; however, participants were excluded if they met criteria for a current alcohol use disorder or had a history of alcohol use disorder within the past year, had a lifetime history of more than 15 alcohol-related blackouts, met criteria for more than mild cannabis use disorder or reported regular cannabis use before age 15 years. All patients were medication-free for at least 2 weeks for all antidepressants (3 months for dopaminergic, 6 weeks for fluoxetine, 8 weeks for neuroleptics, 2 weeks for benzodiazepines or any anxiolytic and 2 weeks for any other antidepressants). Use of medications, vitamins or herbal supplements was assessed at screening, and such treatments were not permitted during study participation. Of the 155 participants assessed for eligibility, 57 completed the biomarker assessment baseline session and could be randomized, but we lost contact with 3 participants before they could receive medication. Three additional participants failed predetermined PRT task quality checks, and a biomarker could not be determined for them. Three participants started medication treatment but dropped out before completing the first follow-up (week 1) assessment.

### BUP and SER treatments

Both BUP and SER are common ‘on-label’ Food and Drug Administration-approved antidepressant medications, with established safety and efficacy profiles. SER is an SSRI, while BUP is an atypical antidepressant. While BUP is generally considered safe at therapeutic doses, overdose can lead to vascular effects, including hypertension and tachycardia. Therefore, we screened for ECG abnormalities before enrollment, and study physicians assessed participants for increases in blood pressure, symptoms of mania, psychosis or other neuropsychiatric reactions. The starting dose of SER was 50 mg, titrated in increments of 50 mg up to the target dose of 200 mg. The starting dose of BUP was 150 mg, titrated in increments of 150 mg to the target dose of 450 mg. To preserve double-blinding, dose adjustments were ordered at identical, protocol-defined time points for all participants (baseline, week 1 and week 2), regardless of treatment assignment, on the basis of tolerability. Not all participants reached the target dose, with some completing treatment with a lower dose. Although BUP reaches its maximum dose earlier than SER, study physicians and research staff continued to order protocol-consistent dose adjustments at each scheduled titration point for all participants. The actual dose implementation was managed by the research pharmacy, which alone had access to treatment assignment and ensured participants remained at the appropriate maximum dose when reached (that is, no further dose increase for BUP past week 2). Therefore, neither the physician nor the experimenters were aware of the current dose, which could have potentially allowed to infer the drug type. Further, blinding was maintained by overcapsulating commercially available tablets. Side effects were monitored at each weekly visit by the study physician (see scales below). The average dose for those prescribed BUP was 378.6 mg (90.2) and the average dose for those prescribed SER was 148.1 mg (47.0).

The total treatment period for the study was 8 weeks, and participants met intent-to-treat criteria if they completed at least 1 week of treatment. We argue that it is valuable to include participants who dropped out early given that they may have experienced side effects or did not tolerate the drug for other reasons, thus suggesting the medication was not suitable for them even though efficacy cannot be adequately determined after 1 week of treatment. In a double-blind design, clinicians, research assistants and researchers interacting with participants were blinded with respect to both biomarker and treatment assignment. Importantly, because the number and timing of protocol-ordered dose adjustments did not differ between treatment arms, titration procedures could not be used to infer treatment assignment. Because participants were blind to the medication they received, they were informed of all the common side effects of either drug. Study biostatistician (B.R.)—who did not interact with study participants—assessed the biomarker stratification mid-way through the trial to evaluate that biomarker distribution was consistent with EMBARC, that is, an approximately equal proportion of participants were biomarker positive for each drug. Separate clinical interviewers (blind to biomarker status or drug assignment) administered the clinical scales used to evaluate clinical response independent of study psychiatrists.

### Adverse effects

Frequency, Intensity, Burden of Side Effects Rating (FIBSER) and Concise Health Risk Tracking (CHRT) were used by the study psychiatrists to monitor side effects and dosing adjustment. Two participants receiving BUP and three participants receiving SER experienced side effects and withdrew.

### Primary outcomes

A primary outcome of the EMBARC study was the HRSD, 17-item version. Following our preregistration at ClinicalTrials.gov, the primary outcome of the SMART-D study (determined by the Wellcome Leap MC Psych Consortium) was the MADRS, with response defined as 50% change on both scales. Remission was defined as a MADRS score of ≤10. MADRS^21^ and HRSD^22^ were administered to participants by trained clinicians. The 10-item MADRS was administered eight times across the study, and the 17-item HRSD was administered at baseline in SMART-D.

### Other clinical assessments

The 12-item 5-point Likert scale on Neuroticism Five-Factor Inventory^23^ was used to examine participants’ self-reported neuroticism. The measure was collected at baseline only. The 14-item SHAPS^24^ self-report measure was administered nine times across the study to assess participants’ anhedonia. These items were assessed on a 4-point Likert scale.

### Neurocognitive tasks

The PRT^25^^,26^ and Eriksen Flanker Task^27,28^ were administered to extract the relevant neurocognitive parameters to assess indication status for BUP and SER markers, respectively. Both tasks were administered as implemented in the EMBARC trial^5,7^, counterbalanced in order across participants, using E-Prime software (version 2.0, Neurobehavioral Systems). Participant’s responses were collected via a response pad (RB-740, Cedrus Corporation), and stimuli were displayed on a 22.5-inch VIEWPixx monitor (VPixx Technologies) at a viewing distance of 50 cm. Data were collected while also recording 96-channel EEG. However, the EEG data were not used in the predictive algorithm and will be reported elsewhere. Details about the task design of the Eriksen Flanker task, and PRT, including response bias and computational modeling analysis of the PRT, are provided in Supplementary Information.

### MRI data acquisition

Structural and functional images were acquired on a 3-T Siemens Prisma-Fit system using a 32-channel head coil. A T1-weighted anatomical image was acquired using a three-dimensional MPRAGE sequence (repetition time of 2,400 ms, echo time of 2.22 ms, inversion time of 1,000 ms and flip angle of 8°) with 0.8-mm isotropic voxels (field of view of 256 mm, sagittal orientation, 208 slices). Parallel imaging used GRAPPA with a phase-encoding acceleration factor of 2; phase-encoding was anterior to posterior. A total of 300 BOLD images were acquired using a multi-echo gradient-echo echo planar imaging sequence (repetition time of 1.33 s, four echoes with echo times of 12.6, 29.16, 45.72 and 62.28 ms, flip angle of 67°, 60 axial slices, 2.5-mm isotropic voxels, multiband factor of 4, GRAPPA of 2, phase-encoding anterior to posterior and field of view of 216 mm × 216 mm). A brief reverse-phase (posterior to anterior) run in addition to the anterior-to-posterior run was acquired for TOPUP-based distortion correction.

### MRI data preprocessing

We adapted our MRI processing pipeline to harmonize the multi-echo data acquired in SMART-D with the CONN toolbox pipelines (version 21.a, Matlab R2022a) run on the EMBARC data to ensure rsFC marker generalizability across studies. First, we ran the meica.py^29^ on the four individual echo trains to obtain the optimally combined echo images. We then ran the default CONN pipeline. Specifically, CONN uses SPM12 and includes realignment, normalization in Montreal Neurological Institute (MNI) space and smoothing with a 6-mm kernel. For motion correction and denoising, CONN uses SPM12 to assess head motion by translation and rotation in *x*, *y* and *z* directions. It then leverages artifact detection tools^30^ to calculate time points of significant head motion with more than 0.5 mm motion compared with the previous frame, or global mean intensity change of more than 3 s.d. from mean intensity across functional scans for each participant. Any participant with >15% outlier volumes out of the resting-state scan series was excluded from group-level analyses. Default CONN parameters were used for the remainder of the pipeline. We then extracted the time series from the rACC region identified by Ang et al.^5^ and from the bilateral NAcc mask in MNI152 space taken from the Harvard–Oxford atlas and correlated those time series to obtain the connectivity marker values.

### BUP and SER biomarkers

We applied the two prediction models developed from the EMBARC dataset, along with their corresponding optimal probability cut-offs, to participants in SMART-D to determine their BUP and SER marker status. For each participant, we extracted the required pretreatment predictors and computed the predicted probabilities of response to BUP and SER. Participants whose predicted probability of BUP response exceeded the optimal threshold were classified as BUP^+^; otherwise, they were classified as BUP^−^. SER^+^ and SER^−^ were defined analogously. These markers reflect whether BUP or SER is expected to be beneficial for a participant based on their pretreatment profile.

### Analysis of treatment responses in SMART-D

We used LMMs to examine the associations between trajectories of MADRS scores over the follow-up period and the factors of interest, as detailed in the preregistered analysis protocol (https://osf.io/tfrz5/). We tested for group differences in MADRS trajectories between (1) consistent versus inconsistent assignment of drug to marker, (2) marker status (both positive, that is, BUP^+^/SER^+^, one positive, that is, BUP^+^/SER^−^ or BUP^−^/SER^+^ and both negative, that is, BUP^−^/SER^−^). We also tested for group differences between participants who received BUP versus SER as treatment, expecting no significant differences in trajectories between these two drugs; this comparison was not preregistered. All models adjusted for age and sex and included time as a continuous variable. For analyses that did not incorporate the BUP or SER markers, the assigned treatment and its interaction with time were included as fixed effects. For analyses involving marker status, the marker (or a derived quantity, such as consistency) and its interaction with time were entered as fixed effects. For analyses of consistent versus inconsistent group status, group status and its interaction with time were included as fixed effects. Each LMM included both a random intercept and a random slope for time to account for within-participant correlations across repeated measures. Hypothesis testing was conducted using Satterthwaite’s approximation^31^ to the denominator degrees of freedom. Chi-squared and Fisher’s exact tests comparing the rates of response and remission between consistent versus inconsistent groups and between marker status groups were also preregistered and conducted here. We also tested for response and remission rates between participants who received BUP versus SER.

### Reporting summary

Further information on research design is available in the Nature Portfolio Reporting Summary linked to this article.

## Supplementary information

Supplementary Information Supplementary Tables 1–5, Figs. 1 and 2 and text.

Reporting Summary

## Data Availability

Source data for SMART-D are available upon reasonable request.

## Code availability

The code used to analyze the data is available via GitHub at https://github.com/peterzhukovsky/smartd.
