# Supplementary material for: A precision medicine trial of bupropion and sertraline for major depressive disorder using a biomarker-guided sequential multiple-assignment design
Source: Nat Ment Health. 2026 Jul 6;4(7):1099–108. doi: 10.1038/s44220-026-00671-z (PMC13341311; doi:10.1038/s44220-026-00671-z)
Supplement: Supplementary file 1 — Supplementary Tables 1–5, Figs. 1 and 2 and text. [file 44220_2026_671_MOESM1_ESM.pdf]

nature mental health

Supplementary information

https://doi.org/10.1038/s44220-026-00671-z

A precision medicine trial of bupropion and sertraline for major depressive disorder using a biomarker-guided sequential multiple-assignment design

In the format provided by the authors and unedited

Supplementary Materials

Table of contents

1. Supplementary Tables
2. Supplementary Figures
3. Supplementary Methods
   3.1. Details on the BUP and SER biomarker models
   3.2. Post-hoc power analyses
   3.1. Eriksen Flanker task and Probabilistic Reward tasks

Supplementary Table 1a. Regression coefficients from the logistic regression models predicting BUP and SER response status.

Predictors	Bupropion Marker Model			Sertraline Marker Model		
	Log-Odds	CI	*p*	Log-Odds	CI	*p*
**Interference Flanker**	-	-	-	-3.69	-8.32 – 0.57	0.101
**Employment Status**	-	-	-	1.54	0.63 – 2.53	0.001
**Neuroticism**	-	-	-	0.03	-0.04 – 0.11	0.397
**HRSD total**	-	-	-	0.03	-0.08 – 0.14	0.641
**Response Bias**	7.14	-0.94 – 18.51	0.129			
**Reward Sensitivity**	3.19	-0.46 – 8.01	0.125			
**NAcc-rACC rsFC**	21.64	7.63 – 48.59	0.03	-3.17	-7.25 – 0.60	0.11

Note. Nacc: nucleus accumbens, rACC: rostral anterior cingulate cortex, rsFC: resting state functional connectivity

Supplementary Table 1b: Estimated standardized regression coefficients from the logistic regression models predicting BUP and SER response status. Predictors were standardized prior to model fitting to facilitate direct comparison of contributions across variables.

**BUP response**					**SER response**				
Predictors	*Estimate*	*SE*	*CI*	*p*	*Predictors*	*Estimate*	*SE*	*CI*	*p*
(Intercept)	-0.63	0.55	-1.83 – 0.38	0.249	(Intercept)	-0.3	0.23	-0.76 – 0.14	0.181
Response bias	1.1	0.73	-0.14 – 2.86	0.129	Inference Flanker	-0.41	0.25	-0.92 – 0.06	0.101
Reward sensitivity	0.99	0.65	-0.14 – 2.49	0.125	Employment status	0.77	0.24	0.31 – 1.26	**0.001**
NAcc-rACC rsFC	3.04	1.40	1.07 – 6.82	**0.03**	NAcc-rACC rsFC	-0.40	0.25	-0.92 – 0.08	0.11
					Neuroticism	0.20	0.23	-0.26 – 0.67	0.397
					HRSD total	0.11	0.25	-0.37 – 0.61	0.641

Supplementary Table 2. Decrease in area under the ROC curve (AUC) after removing each predictor from the full logistic regression model for BUP and SER response status.

**BUP response (Full model AUC = 0.856)**		**SER response (Full model AUC = 0.659)**	
Removed Predictor	*Decrease in AUC*	*Removed Predictor*	*Decrease in AUC*
Response bias	0.006	Inference Flanker	0.014
Reward sensitivity	0.006	Employment status	0.148
NAcc-rACC rsFC	0.097	NAcc-rACC rsFC	0.012
		Neuroticism	0.004
		HRSD total	0.011

Supplementary Table 3. Distribution of different marker status in the EMBARC stage 2 data

	**BUP+**	**BUP-**
**SER+**	8 (10.8%)	31 (41.9%)
**SER-**	14 (18.9%)	21 (28.4%)

Supplementary Table 4. Comparison of rates of participants enrolled in psychotherapy during SMART-D. Fisher's exact test indicated no significant differences between therapy status and group (p=.393).

**Characteristic**	**Group 1 N = 14 BUP+/SER+ *n* (%) or *M* (*SD*)**	**Group 2 N = 17 BUP+/SER- *n* (%) or *M* (*SD*)**	**Group 3 N = 10 BUP-/SER+ *n* (%) or *M* (*SD*)**	**Group 4 N = 7 BUP-/SER- *n* (%) or *M* (*SD*)**	**Fisher's Exact Test Statistic**
**Current Psychotherapy**	6 (43%)	5 (29%)	1 (10%)	2 (29%)	p=.393

Supplementary Table 5. Estimated regression coefficients from the LMM in which participant groups were defined by their BUP and SER markers. The reference group was BUP-/SER-

**Predictors**	Estimates	CI	*p*
sex	-1.49	-5.46 – 2.48	0.462
age	-0.1	-0.33 – 0.14	0.421
Time	-1.18	-2.10 – -0.25	**0.013**
BUP-/SER+	2.84	-3.92 – 9.60	0.409
BUP+/SER-	3.89	-2.36 – 10.13	0.222
BUP+/SER+	4.41	-2.18 – 11.00	0.188
Time:[BUP-/SER+]	-1.35	-2.56 – -0.14	**0.029**
Time:[BUP+/SER-]	-1.26	-2.38 – -0.15	**0.026**
Time:[BUP+/SER+]	-1.04	-2.17 – 0.10	0.073

Supplementary Figure 1. Trajectories of depression scores **(A)** and response rates **(B)** for participants who received sertraline vs. bupropion. Mean MADRS scores are shown at each assessment with 95% confidence intervals based on all observations. MADRS: Montgomery-Asberg Depression Rating Scale. n.s.: not significant

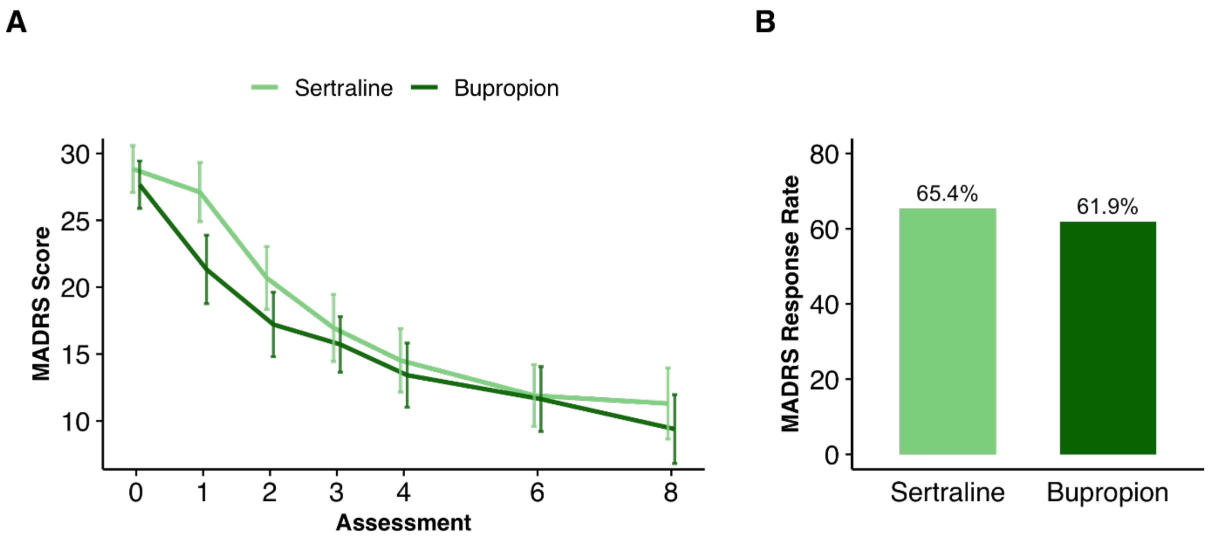

Supplementary Figure 2. Trajectories of depression scores **(A)** and response rates **(B)** for participants across four different marker groups. Mean MADRS scores are shown at each assessment with 95% confidence intervals based on all observations. MADRS: Montgomery-Asberg Depression Rating Scale

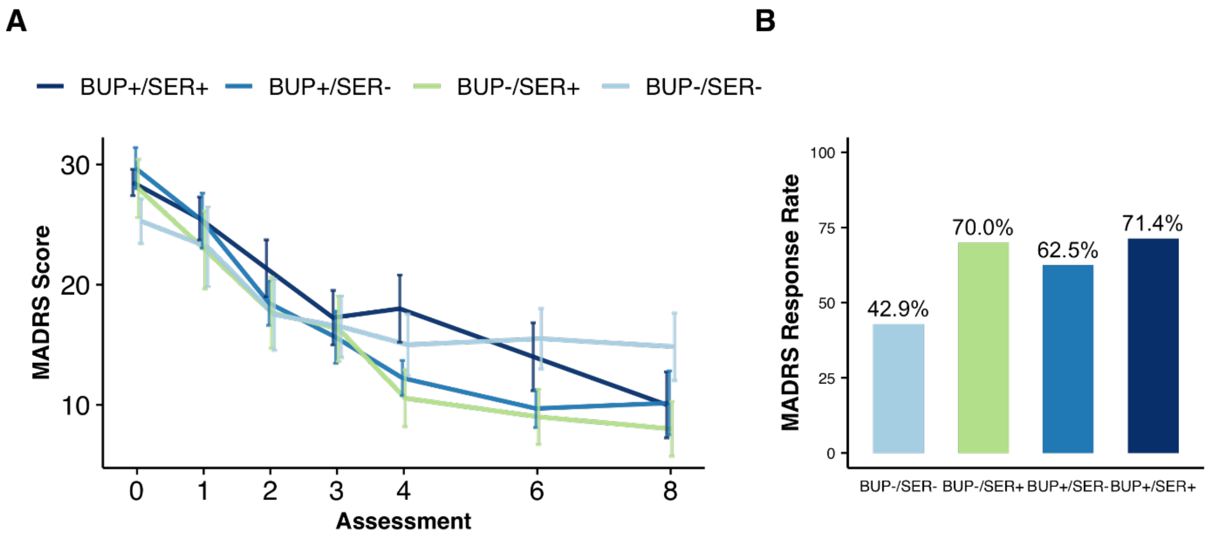

Supplementary Methods

Details on the BUP and SER biomarker models

We considered three model classes: standard logistic regression, LASSO-penalized logistic regression, and random forest when building the biomarker models. Logistic regression served as a parsimonious baseline appropriate for low-dimensional settings, whereas LASSO regularization was included to mitigate overfitting and improve stability in finite samples. Random forest was used as a flexible, nonparametric approach to capture potential nonlinear effects and interactions. For the bupropion model (N=36), performance was assessed using leave-one-out cross-validation, whereas for the sertraline model (N=96), we used repeated 10-fold cross-validation with 1,000 repetitions to obtain stable estimates. Predictive performance was quantified using the area under the receiver operating characteristic curve (AUROC), and the model with the highest cross-validated AUROC was selected and refit on the full dataset to obtain the biomarker model. For LASSO models, the regularization parameter was selected via cross-validation, and for random forest, model complexity was constrained by enforcing a minimum node size of five. Predicted probabilities were converted to binary response indicators using a threshold chosen to minimize the Euclidean distance to (1,1) on the ROC curve (based on the average ROC curve for the sertraline model). Given the low predictor-to-sample ratios (3:36 for bupropion and 5:96 for sertraline), the risk of overfitting for logistic regression is modest; nevertheless, the use of regularization, constrained model complexity, and cross-validation provides additional safeguards and yields robust out-of-sample performance estimates. The binary response indicators were subsequently used as stratification variables in the SMART-D trial randomization.

Post-hoc power analyses

Post hoc power analyses in Gpower (v.3.1) confirmed that a within-between ANOVA with two groups would have low power ($f = 0.10$, power = 0.43) to detect small effects, but high power to detect medium effects ($f = 0.25$, power = 0.99). Similarly, a 3-group analysis would have low power ($f = 0.10$, power = 0.31) to detect small effects, but high power to detect medium effects ($f = 0.25$, power = 0.99). A 4-group analysis would have low power ($f = 0.1$, power = 0.25) to detect small effects, but high power to detect medium effects ($f = 0.25$, power = 0.99).

Eriksen Flanker task and Probabilistic Reward tasks

Eriksen Flanker task. The Eriksen Flanker Task is a widely used neurocognitive task to probe cognitive control, specifically response inhibition, conflict monitoring and error processing. First, participants completed a 30-trial practice (15 congruent, 15 incongruent trials) before the main task. On each trial, flankers appeared alone for 100 ms and were then joined by the central target arrow for an additional 50 ms (total display 150 ms); participants indicated the target's left/right direction via button press, with accuracy and reaction time (RT) recorded. The main task comprised four blocks of 70 trials (46 congruent, 24 incongruent), totaling 280 trials (184 congruent, 96 incongruent). To equate difficulty across individuals, we used an adaptive response window set to the 85th percentile of each participant's incongruent-trial RTs from the

preceding block (block 1 used the practice distribution). Each trial then showed a 1500 ms fixation; if no response occurred by the end of the response window, a “TOO SLOW!” screen appeared for 300 ms, otherwise that interval contained fixation, and a final 200–400 ms jittered fixation ended the trial (overall duration 2050–2250 ms). Congruent/incongruent sequences were generated with optseq2[26] and were identical across participants. Mid-study, brief block-wise feedback was added to stabilize performance: when a block had <3 incongruent errors, the next prompt emphasized speed; when ≥6, it emphasized accuracy; otherwise it emphasized balancing speed and accuracy. Quality control flagged outlier trials if raw RT <150 ms or if log-RT exceeded the participant’s mean ± 3 SD (computed separately by congruency) and excluded datasets with >28 RT outliers (>10%), <160 outlier-free congruent trials, <72 outlier-free incongruent trials, or <50% accuracy in either condition. Flanker interference accuracy, as calculated by mean accuracy in congruent minus mean accuracy in incongruent trials, was used as the neurocognitive parameter extracted from the Erikson Flanker task.

Probabilistic Reward Task (PRT). The PRT is a widely used laboratory measure of reward learning and responsiveness. In each trial, participants discriminated via button press between two briefly presented face stimuli (duration: 100 ms) that differed only in the length of a line indicating the face’s mouth (11.5 vs. 13.0 mm). Each trial began with a fixation cross (750–900 ms), followed by a mouthless face (500 ms), after which the short or long mouth appeared for 100 ms; the face then remained on screen until response. Participants performed 300 trials over three blocks with breaks in between. Across each block, short and long mouths were presented in equal numbers in pseudo-random order with the constraint that the same stimulus did not repeat more than three times consecutively. Unknown to participants, correct responses were reinforced asymmetrically (3:1): one stimulus (“rich”) yielded feedback three times more often than the other (“lean”), with on-screen feedback (“Correct!! You won 20 Cents”) to induce a systematic response bias toward the more frequently rewarded option. Stimulus assignment to rich and lean conditions were counterbalanced.

PRT: Signal Detection Theory Analysis. The PRT administered extended the original EMBARC implementation by adding a third block (300 trials total). However, to ensure comparability with the EMBARC-based algorithm, only the first two blocks (200 trials) were entered into the analyses. As commonly employed for the PRT, performance was analyzed via signal detection theory[24] providing a quantification of response bias, an objective index of reward responsiveness. For quality control, discriminability (a proxy of task difficulty) was also computed and trials were flagged as outliers for reaction times (RT) <150 ms or >2500 ms or if log-RT exceeded a participant’s mean ± 3 SD. Participants were excluded if any of the first two blocks had <80 valid trials, <20 rich rewards or <6 lean rewards, or if the rich:lean reward ratio fell below 2. Response bias as a marker of reward learning was used as one neurocognitive parameter extracted from the PRT.

PRT: Computational Modeling. PRT choices were modeled with an action reinforcement-learning model[5,27]. On each trial, values were learned for the two response options irrespective of stimulus identity, updated by a delta rule with a learning rate (ε), and translated into choices via a softmax that captured reward sensitivity (ρ) as the immediate impact of obtained rewards

on action values. Parameters were estimated in a hierarchical framework and expectation-maximization was used to obtain group-level priors and a Laplace approximation to the individual posteriors for each participant was applied. To ensure well-behaved estimation, ε was fit on a logit scale (0–1) and ρ on a log scale (>0). We used the hierarchical modeling approach with group-level priors and determined cut-offs for treatment assignment based on the EMBARC-sample. Therefore, we included the EMBARC sample data in modeling participant's data in SMART-D. Thus, the individual's data were appended to the original data frame, the action model was refitted and the individuals' reward sensitivity was extracted for the treatment assignment. As such, reward sensitivity (ρ) was used as another neurocognitive parameter extracted from the PRT.

REFERENCES

1. Gaynes BN, Warden D, Trivedi MH, Wisniewski SR, Fava M, Rush AJ. What did STAR*D teach us? Results from a large-scale, practical, clinical trial for patients with depression. *Psychiatr Serv*. 2009;60(11):1439-1445. doi:10.1176/PS.2009.60.11.1439/ASSET/722D5AD1-C40E-4792-BB72-9E7E7E708561/ASSETS/GRAPHIC/JZ07F2.JPEG
2. Papakostas GI, Fava M, Thase ME. Treatment of SSRI-Resistant Depression: A Meta-Analysis Comparing Within- Versus Across-Class Switches. *Biol Psychiatry*. 2008;63(7):699-704. doi:10.1016/j.biopsych.2007.08.010
3. Li J, Chen H, Liao W. Biologically Annotated Heterogeneity of Depression Through Neuroimaging Normative Modeling. *Biol Psychiatry*. Published online 2025. doi:10.1016/J.BIOPSYCH.2025.07.002
4. Laifenfeld D, Albeldas C, Solal TC, McIntyre RS, Stahl S. Toward Precision Psychiatry: Innovations and Prospects in Treating Depression. *Prim care companion CNS Disord*. 2025;27(5). doi:10.4088/PCC.25NR03970
5. Ang YS, Kaiser R, Deckersbach T, et al. Pretreatment Reward Sensitivity and Frontostriatal Resting-State Functional Connectivity Are Associated With Response to Bupropion After Sertraline Nonresponse. *Biol Psychiatry*. 2020;88(8):657-667. doi:10.1016/j.biopsych.2020.04.009
6. Zhukovsky P, Trivedi MH, Weissman M, Parsey R, Kennedy S, Pizzagalli DA. Generalizability of Treatment Outcome Prediction Across Antidepressant Treatment Trials in Depression. *JAMA Netw Open*. 2025;8(3):1-12. doi:10.1001/jamanetworkopen.2025.1310
7. Webb CA, Trivedi MH, Cohen ZD, Dillon DG, Pizzagalli DA. Personalized prediction of antidepressant versus placebo response: Evidence from the EMBARC study. *Psychol Med*. 2019;49(7):1118-1127. doi:10.1017/S0033291718001708.Personalized
8. Poirot MG, Ruhe HG, Mutsaerts HJMM, et al. Treatment Response Prediction in Major Depressive Disorder Using Multimodal MRI and Clinical Data: Secondary Analysis of a Randomized Clinical Trial. *Am J Psychiatry*. 2024;181(3):223-233. doi:10.1176/appi.ajp.20230206
9. Trivedi MH, McGrath PJ, Fava M, et al. ESTABLISHING MODERATORS and BIOSIGNATURES OF ANTIDEPRESSANT RESPONSE IN CLINICAL CARE (EMBARC): Rationale and Design. *J Psychiatr Res*. 2016;78:11–23. doi:10.1016/j.jpsychires.2016.03.001.ESTABLISHING
10. Kelley ME, Choi KS, Rajendra JK, et al. Establishing Evidence for Clinical Utility of a Neuroimaging Biomarker in Major Depressive Disorder: Prospective Testing and Implementation Challenges. *Biol Psychiatry*. 2021;90(4):236-242. doi:10.1016/j.biopsych.2021.02.966
11. van der Vinne N, Vollebregt MA, Rush AJ, Eebes M, van Putten MJAM, Arns M. EEG biomarker informed prescription of antidepressants in MDD: a feasibility trial. *Eur Neuropsychopharmacol*. 2021;44:14-22. doi:10.1016/J.EURONEURO.2020.12.005
12. Hack LM, Jubeir J, Hilton R, et al. A stratified precision medicine trial targeting α2A-adrenergic receptor agonism as a treatment for the cognitive biotype of depression. *Nat Ment Heal 2025 311*. 2025;3(11):1363-1373. doi:10.1038/s44220-025-00510-7
13. Cipriani A, Furukawa TA, Salanti G, et al. Comparative efficacy and acceptability of 21 antidepressant drugs for the acute treatment of adults with major depressive disorder: a systematic review and network meta-analysis. *Lancet*. 2018;391(10128):1357-1366. doi:10.1016/S0140-6736(17)32802-7
14. Cools R, Nakamura K, Daw ND. Serotonin and dopamine: unifying affective, activational, and decision functions. *Neuropsychopharmacology*. 2011;36(1):98-113.

doi:10.1038/npp.2010.121
15. Scholes KE, Harrison BJ, O'Neill B V., et al. Acute Serotonin and Dopamine Depletion Improves Attentional Control: Findings from the Stroop Task. *Neuropsychopharmacol 2007 327*. 2006;32(7):1600-1610. doi:10.1038/sj.npp.1301262
16. Pizzagalli DA. Depression, stress, and anhedonia: Toward a synthesis and integrated model. *Annu Rev Clin Psychol*. 2014;10:393-423. doi:10.1146/annurev-clinpsy-050212-185606
17. Marzi C, Giannelli M, Barucci A, Tessa C, Mascalchi M, Diciotti S. Efficacy of MRI data harmonization in the age of machine learning: a multicenter study across 36 datasets. *Sci Data 2024 111*. 2024;11(1):115-. doi:10.1038/s41597-023-02421-7
18. Chekroud AM, Hawrilenko M, Loho H, et al. Illusory generalizability of clinical prediction models. *Science (80-)*. 2024;383:164-167. doi:10.1126/science.adg8538
19. Chandler GM, Iosifescu D V., Pollack MH, Targum SD, Fava M. RESEARCH: Validation of the Massachusetts General Hospital Antidepressant Treatment History Questionnaire (ATRQ). *CNS Neurosci Ther*. 2010;16(5):322. doi:10.1111/J.1755-5949.2009.00102.X
20. Montgomery A, Asberg M. A New Depression Scale Designed to be Sensitive to Change. *Br J Psychiatry*. 1979;134:382-389.
21. HAMILTON M. Development of a rating scale for primary depressive illness. *Br J Soc Clin Psychol*. 1967;6(4):278-296. doi:10.1111/J.2044-8260.1967.TB00530.X
22. Snaith RP, Hamilton M, Morley S, Humayan A, Hargreaves D, Trigwell P. A scale for the assessment of hedonic tone. The Snaith-Hamilton Pleasure Scale. *Br J Psychiatry*. 1995;167(JULY):99-103. doi:10.1192/bjp.167.1.99
23. Costa PT, McCrae RR. Normal personality assessment in clinical practice: The NEO Personality Inventory. *Psychol Assess*. 1992;4(1):5-13. doi:10.1037//1040-3590.4.1.5
24. Pizzagalli DA, Jahn AL, O'Shea JP. Toward an objective characterization of an anhedonic phenotype: a signal-detection approach. *Biol Psychiatry*. 2005;57(4):319-327. doi:10.1016/J.BIOPSYCH.2004.11.026
25. Eriksen BA, Eriksen CW. Effects of noise letters upon the identification of a target letter in a nonsearch task. *Percept Psychophys*. 1974;16(1):143-149. doi:10.3758/BF03203267/METRICS
26. Dale AM. Optimal experimental design for event-related fMRI. *Hum Brain Mapp*. 1999;8(2-3):109. doi:10.1002/(sici)1097-0193(1999)8:2/3<109::aid-hbm7>3.0.co;2-w
27. Huys QJ, Pizzagalli DA, Bogdan R, Dayan P. Mapping anhedonia onto reinforcement learning: a behavioural meta-analysis. *Biol Mood Anxiety Disord*. 2013;3(1). doi:10.1186/2045-5380-3-12
28. Kundu P, Brenowitz ND, Voon V, et al. Integrated strategy for improving functional connectivity mapping using multiecho fMRI. *Proc Natl Acad Sci U S A*. 2013;110(40):16187-16192. doi:10.1073/PNAS.1301725110/SUPPL_FILE/PNAS.201301725SI.PDF
