# Supplementary material for: A precision medicine trial of bupropion and sertraline for major depressive disorder using a biomarker-guided sequential multiple-assignment design
Source: Nat Ment Health. 2026 Jul 6;4(7):1099–108. doi: 10.1038/s44220-026-00671-z (PMC13341311; doi:10.1038/s44220-026-00671-z)
Supplement: Supplementary file 2 — Reporting Summary [file 44220_2026_671_MOESM2_ESM.pdf]

nature portfolio

Corresponding author(s): Diego A. Pizzagalli
Last updated by author(s): May 12, 2026

Reporting Summary

Nature Portfolio wishes to improve the reproducibility of the work that we publish. This form provides structure for consistency and transparency in reporting. For further information on Nature Portfolio policies, see our Editorial Policies and the Editorial Policy Checklist.

Statistics

For all statistical analyses, confirm that the following items are present in the figure legend, table legend, main text, or Methods section.

n/a	Confirmed	
☐	☒	The exact sample size (*n*) for each experimental group/condition, given as a discrete number and unit of measurement
☐	☒	A statement on whether measurements were taken from distinct samples or whether the same sample was measured repeatedly
☐	☒	The statistical test(s) used AND whether they are one- or two-sided<br>*Only common tests should be described solely by name; describe more complex techniques in the Methods section.*
☐	☒	A description of all covariates tested
☐	☒	A description of any assumptions or corrections, such as tests of normality and adjustment for multiple comparisons
☐	☒	A full description of the statistical parameters including central tendency (e.g. means) or other basic estimates (e.g. regression coefficient) AND variation (e.g. standard deviation) or associated estimates of uncertainty (e.g. confidence intervals)
☐	☒	For null hypothesis testing, the test statistic (e.g. F, t, r) with confidence intervals, effect sizes, degrees of freedom and P value noted<br>*Give P values as exact values whenever suitable.*
☒	☐	For Bayesian analysis, information on the choice of priors and Markov chain Monte Carlo settings
☐	☒	For hierarchical and complex designs, identification of the appropriate level for tests and full reporting of outcomes
☐	☒	Estimates of effect sizes (e.g. Cohen's *d*, Pearson's *r*), indicating how they were calculated

Our web collection on statistics for biologists contains articles on many of the points above.

Software and code

Policy information about availability of computer code

Data collection	MRI data were collected on a Siemens PRISMA 3T scanner. The Probabilistic Reward Task (PRT) and Eriksen Flanker Task were administered to extract the relevant neurocognitive parameters to assess indication status for bupropion and sertraline markers, respectively. Both tasks were administered as implemented in the EMBARC trial, counterbalanced in order across participants, using E-Prime software (vers. 2.0, Neurobehavioral systems). Participant’s responses were collected via a response pad (RB-740, Cedrus Corporation, San Pedro, CA) and stimuli were displayed on a on 22.5-inch VIEWPixx monitor (VPixx Technologies, Saint-Bruno, QC, Canada) at a viewing distance of 50 cm. Data were collected while also recording 96-channel EEG. However, the EEG data were not used in the predictive algorithm and will be reported elsewhere.
Data analysis	Data analysis and visualizations were conducted in R (including packages haven, glmnet, pROC, readxl, dplyr)<br>All code used in data analysis is deposited at https://github.com/peterzhukovsky/smartd<br><br>fMRI data was processed using meica.py (built on AFNI software) to obtain the optimally combined echo train and then processed in a harmonized protocol with the EMBARC data using SPM12 CONN toolbox v 21a (v. MATLAB R2022a)

For manuscripts utilizing custom algorithms or software that are central to the research but not yet described in published literature, software must be made available to editors and reviewers. We strongly encourage code deposition in a community repository (e.g. GitHub). See the Nature Portfolio guidelines for submitting code & software for further information.

Data

Policy information about availability of data

All manuscripts must include a data availability statement. This statement should provide the following information, where applicable:

- Accession codes, unique identifiers, or web links for publicly available datasets
- A description of any restrictions on data availability
- For clinical datasets or third party data, please ensure that the statement adheres to our policy

The data used in the present work are available upon reasonable request to the corresponding author.

Research involving human participants, their data, or biological material

Policy information about studies with human participants or human data. See also policy information about sex, gender (identity/presentation), and sexual orientation and race, ethnicity and racism.

Reporting on sex and gender	In both studies from which the present data came, participant Sex identification was based on participant self-report of sex assigned at birth (i.e., female or male) at point of enrollment.<br><br>Neither sex nor gender were used in the analysis given the limited sample size, though we correct for .
Reporting on race, ethnicity, or other socially relevant groupings	In both studies participant racial identification was made via self-report. In EMBARC, participants were asked to indicate their racial identity as "White", "African-American" or "Other". In SMART-D, participants were asked to indicate their racial identity as: "Alaska Native", "Asian", "Black or African American", "Native American", "Pacific Islander", "Some Other Race", "White", "Don't know", or "Prefer not to Answer".<br><br>Both studies also asked participants to self-report on ethnicity, specifically with regard to identifying as hispanic/latine whereby participants were asked: "Do you consider yourself to be Hispanic or Latino?"<br><br>As with Sex and Gender, we have no theoretical expectation that these groupings would be relevant to the outcome, and so they were not examined in the present analysis.
Population characteristics	Population characteristics are provided in Extended Data Table 1.
Recruitment	Biomarker search in EMBARC was a secondary analysis of data collected across several sites and did not involve recruitment specifically conducted for the present study. EMBARC recruited participants at four university sites between August 2011, and December 2015.<br>SMART-D was a novel prospective biomarker-guided RCT, recruiting treatment-seeking individuals from the Greater Boston Community. Participants were recruited from the community using word-of-mouth, social media and public transport advertisements, and notably BuildClinical recruitment services.
Ethics oversight	For EMBARC, the Institutional Review Board of each of the five sites individually approved the procedures. The Massachusetts General Brigham Institutional Review Board approved the procedures of SMART-D, and all participants provided written informed consent prior to participating in their respective studies. All participants were compensated for completing the study sessions.

Note that full information on the approval of the study protocol must also be provided in the manuscript.

Field-specific reporting

Please select the one below that is the best fit for your research. If you are not sure, read the appropriate sections before making your selection.

☒ Life sciences ☐ Behavioural & social sciences ☐ Ecological, evolutionary & environmental sciences

For a reference copy of the document with all sections, see nature.com/documents/nr-reporting-summary-flat.pdf

Life sciences study design

All studies must disclose on these points even when the disclosure is negative.

Sample size	Total analyzed data from SMART-D: n=47 participants<br>Total analyzed data from EMBARC: stage 1 n=96; stage 2 n=36.<br><br>The original power analyses used n=30 per group, and considered exclusively EMBARC data, which provided the backbone and starting point for our prospective RCT. We revised power analyses after the midpoint of the SMART trial, when it became evident that the distribution of the markers in our prospective sample was (as one could expect) not identical to the EMBARC study, which included four recruiting sites across the USA. In addition, the funding agency (Wellcome Leap) did not allow a no-cost extension, which limited the recruitment period. Despite extensive efforts to achieve a large sample during the available recruitment window, enrollment remained challenging due to the study's strict inclusion and exclusion criteria.

	We include a new post hoc power calculation with our sample (n = 47 across the 4 groups) in the Supplement.
Data exclusions	Data were excluded from participants without MRI or cognitive test data in EMBARC and SMART-D.
Replication	The present study does not include a direct replication of its main analyses.
Randomization	In EMBARC participants were randomized to sertraline or placebo in stage 1 (double blinded); the non-responders to an 8-week trial were switched to bupropion from sertraline or to sertraline from placebo, under double blinded conditions.<br>In SMART-D, participants were randomized to bupropion or sertraline (1:1), stratified by their biomarker status. which assigned 50% of the BUP-marker positive patients to bupropion and 50% of them to sertraline (and vice versa for the SER-marker positive patients).<br>Randomization was stratified by Flanker interference, employment, neuroticism, Hamilton Depression Rating Scale (HDRS), PRT response bias and reward sensitivity and accumbens-rostral ACC connectivity.
Blinding	Both EMBARC and SMART-D were double blinded clinical trials. Stage 1 of EMBARC was placebo controlled, while in stage 2 of EMBARC non-responders to an 8-week trial were switched to bupropion from sertraline or to sertraline from placebo, under double blinded conditions.

Reporting for specific materials, systems and methods

We require information from authors about some types of materials, experimental systems and methods used in many studies. Here, indicate whether each material, system or method listed is relevant to your study. If you are not sure if a list item applies to your research, read the appropriate section before selecting a response.

Materials & experimental systems

n/a	Involved in the study	
☒	☐	Antibodies
☒	☐	Eukaryotic cell lines
☒	☐	Palaeontology and archaeology
☒	☐	Animals and other organisms
☐	☒	Clinical data
☒	☐	Dual use research of concern
☒	☐	Plants

Methods

n/a	Involved in the study	
☒	☐	ChIP-seq
☒	☐	Flow cytometry
☐	☒	MRI-based neuroimaging

Clinical data

Policy information about clinical studies

All manuscripts should comply with the ICMJE guidelines for publication of clinical research and a completed CONSORT checklist must be included with all submissions.

Clinical trial registration	The present work was a secondary analysis of data collected for two other studies - Study 1 (NCT04239963) and Study 2 (NCT05537584)
Study protocol	The protocols relevant to the present work are described in the Methods section of the manuscript. More detailed descriptions of the full protocols can be found at the ClinicTrials.gov listing for each Study (SMART-D: NCT05537584; EMBARC: NCT01407094).
Data collection	Data collection for SMART-D was carried out the McLean Hospital Belmont, Massachusetts between October 27, 2022, and August 26, 2025.
Outcomes	Primary outcomes of the EMBARC study were the Hamilton Depression Rating Scale (HDRS-17). Following our pre-registration, the primary outcome of the SMART-D study (determined by the Wellcome Leap MC Psych Consortium) was the Montgomery-Asberg Depression Rating Scale (MADRS), with response defined as 50% change on both scales. Remission was defined as a MADRS score of ≤ 10. MADRS20 and HDRS21 were administered to participants by trained clinicians. The 10-item MADRS was administered 8 times across the study and the 17-item HDRS was administered at baseline in SMART-D. The 14-item Snaith Hamilton Pleasure Scale (SHAPS22) self-report measure was administered 9 times across the study to assess participants' anhedonia. These items are assessed on a 4-point Likert scale.<br>Secondary outcomes of this preregistered trial are brain measures of anhedonia and are not reported here.

Plants

Seed stocks	*Report on the source of all seed stocks or other plant material used. If applicable, state the seed stock centre and catalogue number. If plant specimens were collected from the field, describe the collection location, date and sampling procedures.*
Novel plant genotypes	*Describe the methods by which all novel plant genotypes were produced. This includes those generated by transgenic approaches, gene editing, chemical/radiation-based mutagenesis and hybridization. For transgenic lines, describe the transformation method, the number of independent lines analyzed and the generation upon which experiments were performed. For gene-edited lines, describe the editor used, the endogenous sequence targeted for editing, the targeting guide RNA sequence (if applicable) and how the editor was applied.*
Authentication	*Describe any authentication procedures for each seed stock used or novel genotype generated. Describe any experiments used to assess the effect of a mutation and, where applicable, how potential secondary effects (e.g. second site T-DNA insertions, mosiacism, off-target gene editing) were examined.*

Magnetic resonance imaging

Experimental design

Design type	resting state
Design specifications	6:39 minutes of multi-echo resting state fMRI
Behavioral performance measures	N/A

Acquisition

Imaging type(s)	functional
Field strength	3T
Sequence & imaging parameters	300 BOLD images were acquired using a multi-echo gradient-echo EPI sequence: TR=1.33 s; four echoes TE=12.6/29.16/45.72/62.28 ms; flip angle=67°; 60 axial slices; 2.5 mm isotropic voxels; multiband factor=4; GRAPPA=2; phase-encoding anterior→posterior, AP; FOV= 216×216 mm
Area of acquisition	whole brain

Diffusion MRI ☐ Used ☒ Not used

Preprocessing

Preprocessing software	First, we ran the meica.py on the four individual echo trains to obtain the optimally combined echo images. We then ran the default CONN pipeline (version 21.a, Matlab R2022a) which is build upon SPM12.
Normalization	Structural images were segmented and spatially normalized (non-linear) to MNI space using SPM12's unified segmentation approach, and the resulting deformation fields were applied to the functional images.
Normalization template	ICBM 152 MNI template
Noise and artifact removal	Motion-related artefacts were identified using Artifact Detection Tools (ART), with volumes exceeding 0.5 mm framewise displacement or 3 standard deviations in global signal intensity flagged as outliers and included as nuisance regressors (scrubbing). Denoising was performed in CONN using regression of six motion parameters and default nuisance covariates (white matter and cerebrospinal fluid signals), followed by band-pass temporal filtering (0.008–0.09 Hz) using CONN's default settings.
Volume censoring	Motion-related artefacts were identified using Artifact Detection Tools (ART), with volumes exceeding 0.5 mm framewise displacement or 3 standard deviations in global signal intensity flagged as outliers and included as nuisance regressors (scrubbing)

Statistical modeling & inference

Model type and settings	ROI-to-ROI functional connectivity was estimated using a general linear model (GLM) implemented in CONN, based on Fisher z-transformed Pearson correlation coefficients between regional time series.
Effect(s) tested	N/A; Resting state ROI-to-ROI connectivity was extracted only

Specify type of analysis: ☐ Whole brain ☒ ROI-based ☐ Both

Anatomical location(s)	The rostral ACC region identified by Ang et al (Biol Psych 2020) and from the bilateral nucleus accumbens mask in MNI152 space taken from the Harvard-Oxford atlas

Statistic type for inference
(See Eklund et al. 2016)

N/A

Correction

N/A

Models & analysis

n/a	Involved in the study
☐	☒ Functional and/or effective connectivity
☒	☐ Graph analysis
☒	☐ Multivariate modeling or predictive analysis

Functional and/or effective connectivity

Pearson correlation between regional BOLD time series
